# Hydrogen sulfide donor sodium hydrosulfide modulates ovarian steroidogenesis and follicular integrity in a DHEA-induced rat model of polycystic ovary syndrome

**DOI:** 10.3389/fendo.2026.1850337

**Published:** 2026-06-19

**Authors:** Aysun Ozbay Onal, Nadiye Koroglu, Alev Cumbul, Engin Sumer, Kubra Acikalin Coskun

**Affiliations:** 1 Istanbul Aydın University, Faculty of Medicine, Department of Histology and Embryology, Istanbul, Türkiye; 2 Acıbadem University, Faculty of Medicine, Department of Obstetrics and Gynecology, Istanbul, Türkiye; 3 Yeditepe University, Faculty of Medicine, Department of Histology and Embryology, Istanbul, Türkiye; 4 Yeditepe University, Faculty of Medicine, Experimental Research Center, Istanbul, Türkiye; 5 Istanbul Aydın University, Faculty of Medicine, Department of Medical Biology, Istanbul, Türkiye

**Keywords:** apoptosis, hydrogen sulfide, NaHS, PCOS, steroidogenesis

## Abstract

**Background:**

Hydrogen sulfide (H_2_S) has emerged as a potential regulator of ovarian physiology; however, its role in polycystic ovary syndrome (PCOS) remains incompletely understood. This study aimed to investigate the effects of the H_2_S donor sodium hydrosulfide (NaHS) on ovarian steroidogenesis, apoptosis and follicular remodelling in a dehydroepiandrosterone (DHEA)-induced rat model of PCOS.

**Methods:**

Thirty adult female Wistar rats were randomly assigned to control, vehicle, PCOS, NaHS, and PCOS+NaHS groups (n=6 each). PCOS was induced by daily subcutaneous injections (s.c) of DHEA (6 mg/100 g body weight) for 20 days. NaHS (200 μg/kg/day) was administered intraperitoneally (i.p.), either alone or in combination with DHEA. Serum hormone levels were analyzed, estrous cyclicity was tracked, and ovarian tissues were examined histologically for follicular development, apoptosis, and the expression of key steroidogenic enzymes (StAR, 3β-HSD, CYP19A1) and H_2_S-producing enzymes (CBS, CTH) at both protein and mRNA levels.

**Results:**

DHEA treatment resulted in disrupted estrous cyclicity, elevated serum estradiol and progesterone levels, reduced numbers of primordial, primary, Graafian follicles and corpora lutea, and increased cystic and atretic follicles. Apoptotic activity and the expression of StAR, 3β-HSD, and CYP19A1 were significantly elevated, whereas CBS and CTH expression levels were reduced. NaHS administration attenuated DHEA-induced alterations by improving estrous cyclicity, reducing follicular damage and apoptosis, downregulating steroidogenic enzyme overexpression, and partially restoring CBS and CTH expression.

**Conclusions:**

Pharmacological supplementation with the H_2_S donor NaHS was associated with modulation of steroidogenic enzyme expression, apoptotic activity, and follicular architecture in a DHEA-induced PCOS model. These findings suggest a potential regulatory interaction between H_2_S-related pathways and ovarian dysfunction in PCOS, warranting further mechanistic investigation.

## Introduction

Polycystic Ovary Syndrome (PCOS) is a heterogeneous endocrine and metabolic disorder affecting women of reproductive age and is characterized by hyperandrogenism, ovulatory dysfunction, and polycystic ovarian morphology (1). Although the exact etiology remains incompletely understood, current evidence suggests that PCOS involves complex interactions among hypothalamic–pituitary–ovarian axis dysregulation, hyperandrogenism, insulin resistance, altered gonadotropin secretion, and abnormal folliculogenesis (2). Increased GnRH pulse frequency favors luteinizing hormone (LH) secretion over follicle-stimulating hormone (FSH), resulting in an elevated LH/FSH ratio in many patients with PCOS. Elevated LH levels stimulate ovarian theca cells to produce excess androgens, whereas relatively insufficient FSH activity impairs granulosa cell function and aromatase activity, thereby disrupting follicular maturation and ovulation. Consequently, follicular growth is arrested, dominant follicle selection is impaired, and chronic anovulation develops (3).

Gasotransmitters are endogenously produced intracellular signaling molecules that regulate multiple physiological functions. Among them, hydrogen sulfide (H_2_S) acts as a gaseous signaling mediator. H_2_S exhibits diverse physiological and pathological effects in several organ systems, including the nervous, urinary, respiratory, cardiovascular, and digestive systems (4, 5). Three key enzymes are responsible for the enzymatic biosynthesis of H_2_S in various cells and tissues: cystathionine γ-lyase (CSE or CTH), cystathionine β-synthase (CBS), and 3-mercaptopyruvate sulfurtransferase (3-MST) (5). The expression of CSE and CBS is tissue-specific and has been identified in the uterus, placenta, and fetal membranes of pregnant rats, as well as in the uteri of non-pregnant rats. In human tissues, CBS and CSE have been expressed in the myometrium, amnion, chorion, and placenta. During pregnancy, their upregulation contributes to local H_2_S production which maintains uterine quiescence and prevents excessive contractions (6). Experimental studies have shown that CBS deficiency is associated with impaired fertility, altered estrous cyclicity, and reduced follicular development in mice (7)In addition, H_2_S-producing enzymes have been identified in murine and porcine oocytes, and inhibition of these enzymes suppresses meiotic maturation, whereas exogenous H_2_S donors such as NaHS accelerate oocyte maturation (8, 9). H_2_S has recently emerged as an important regulator of ovarian physiology and has been implicated in follicular maturation, ovulation, and oocyte meiosis through modulation of oxidative balance, mitochondrial function, and inflammatory signaling pathways. Previous studies also demonstrated that the H_2_S-producing system may participate in ovulatory processes through the regulation of ovulation-related mediators and proteolytic enzymes involved in follicular rupture (10).

Furthermore, limited evidence from experimental PCOS models suggests that impaired H_2_S metabolism and decreased activity of H_2_S-producing enzymes may contribute to oxidative imbalance and ovarian dysfunction, while exogenous H_2_S supplementation may partially ameliorate these alterations (11). Nevertheless, the potential role of H_2_S supplementation in the regulation of ovarian steroidogenic activity, follicular integrity, and apoptotic alterations within the context of PCOS remains insufficiently elucidated. In particular, the relationship between the H_2_S-generating system and steroidogenic enzyme expression in PCOS-associated ovarian pathology has not been comprehensively characterized. Therefore, the present study aimed to investigate the effects of the H_2_S donor sodium hydrosulfide (NaHS) on ovarian morphology, follicular dynamics, apoptosis, and steroidogenic enzyme expression in a DHEA-induced rat model of PCOS.

## Materials and methods

The study was conducted using 30 adult female Wistar albino rats (10 weeks old) obtained from the Experimental Research Application and Research Center of Yeditepe University. All animals were housed individually in cages under controlled environmental conditions (12-hour light/dark cycle, 24 ± 2 °C temperature, 50–60% relative humidity) and were fed a standard pellet diet ad libitum. All experimental procedures were performed in accordance with established standards for animal welfare and ethical use. The study was approved by the Yeditepe University Animal Experiments Ethics Committee (Approval No: 2022/04-2, Date: April 28, 2022).

### Experimental groups and induction of the PCOS model

**Figure 1:**
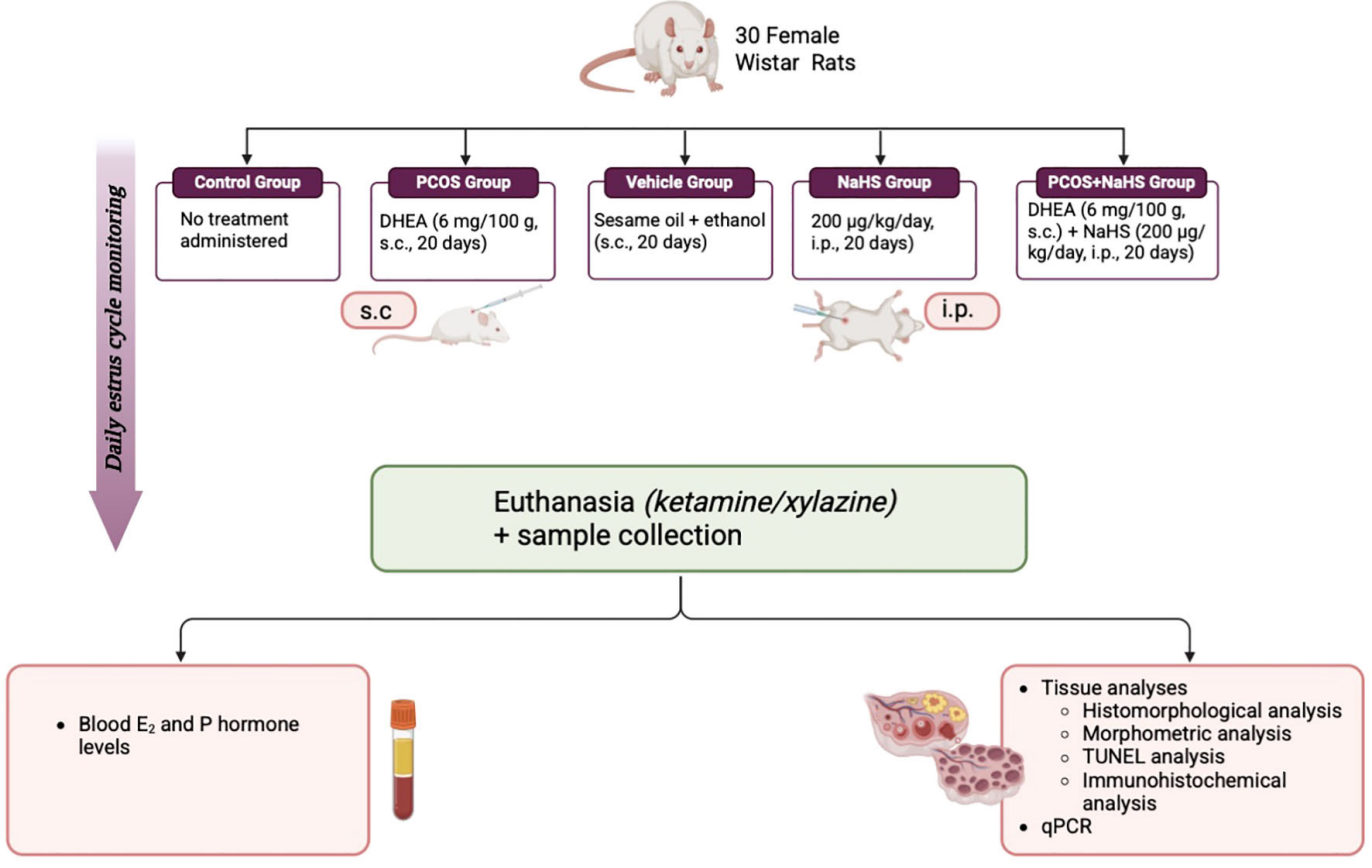
Outline of the experimental design and treatment timeline.

Animals were randomly allocated into experimental groups using a simple randomization method. Regarding sample size determination, a formal power analysis could not be performed due to insufficient and inconsistent variance data available in the literature for the DHEA-induced PCOS model. Therefore, sample size was determined according to the Resource Equation Method in compliance with the 3R principle (Reduction). 30 rats were randomly divided into five experimental groups, each consisting of six rats. All treatments were initiated during the estrus phase. The control group received no treatment. To induce a PCOS model, rats in the PCOS group were administered DHEA (6 mg/100 g body weight; Santa Cruz, catalog no: sc-202573) dissolved in 0.01 mL of 95% ethanol and mixed with 0.09 mL of sesame oil (12, 13). This mixture was administered subcutaneously for 20 consecutive days. The vehicle group received subcutaneous (s.c.) of the same ethanol–sesame oil mixture without DHEA. The sodium hydrosulfide (NaHS) group received daily intraperitoneal (i.p.) injections of NaHS (200 μg/kg/day; Sigma-Aldrich, catalog no: 161527-5G) dissolved in sterile saline (14). In the PCOS+NaHS group, DHEA and NaHS were administered concurrently throughout the experimental period, with NaHS given intraperitoneally two hours after each daily subcutaneous DHEA injection. The dosages and administration routes for DHEA and NaHS were selected based on previously validated *in vivo* studies demonstrating their efficacy and safety (12–14). The selected NaHS dose was considered biologically relevant for modulating H_2_S signaling in reproductive tissues without inducing systemic toxicity. All injections and routine handling procedures were performed at the same time of day by the same investigator under standardized environmental conditions. The experimental design and treatment timeline are schematically illustrated in **Figure 1**.

### Experimental procedures

Throughout the experiment, daily vaginal cytology was performed to monitor reproductive status and confirm estrous cycle disruption in DHEA-treated rats. In the control, vehicle, and NaHS groups, animals were euthanized upon entry into the estrus phase. In contrast, DHEA-treated rats exhibited persistent diestrus or acyclic cytological patterns, consistent with the established phenotype of this model. Accordingly, euthanasia in the PCOS group was performed after confirmation of a stable abnormal cytological pattern maintained for at least 2–3 consecutive days (between days 20 and 22 of treatment). Because persistent diestrus/acyclicity represents a defining characteristic of the DHEA-induced PCOS phenotype, strict phase-matching at euthanasia was not biologically feasible in this group. Thus, tissue collection was conducted under stable and reproducible cytological conditions appropriate for each experimental group. The animals were anesthetized with intraperitoneal xylazine (5 mg/kg) and ketamine HCl (70 mg/kg). After confirming deep anesthesia and complete loss of reflexes, intracardiac blood samples were collected. Euthanasia was then performed using high-concentration carbon dioxide (CO_2_) inhalation. CO_2_ was introduced gradually until respiratory arrest occurred, and exposure was continued for an additional 1–2 minutes to ensure death. Absence of heartbeat and corneal reflex was verified before tissue collection. Bilateral oophorectomy was subsequently performed. The right ovaries were fixed in 10% neutral buffered formalin for histopathological, immunohistochemical, and TUNEL analyses, while the left ovaries were stored at −80 °C for RT-qPCR analysis.

### Serum hormone levels

Serum samples were collected at baseline (day 0, prior to DHEA administration) and at the end of the experimental period (day 20) from the same animals for the determination of estradiol (E2) and progesterone levels. Measurements were performed using commercial ELISA kits (BT Lab; E2: Cat. No. EA0011Ra, Progesterone: Cat. No. EA0063Ra) following the manufacturer’s instructions.

### Vaginal cytology

Vaginal smears were collected daily for 20 days using cotton swabs moistened with physiological saline. The samples were gently spread onto clean glass slides, air-dried, and fixed with 85% ethanol. Subsequently, they were stained with Giemsa solution (Merck) for 20 minutes according to the manufacturer’s protocol. Vaginal smears were collected daily at approximately 08:00 a.m., and the sampling time was kept consistent throughout the experimental period. Estrous cycle stages were determined under a light microscope according to the criteria defined by Cora et al. (15).

### Histopathological analyses

Ovarian tissues were fixed, embedded in paraffin, and sectioned serially at a thickness of 5 µm throughout the entire ovary. All obtained sections were stained with hematoxylin and eosin (H&E) for histopathological evaluation and follicle assessment. Periodic Acid–Schiff (PAS) staining was additionally performed to evaluate the structural integrity of the zona pellucida.

H&E-stained sections were examined under a light microscope for follicular cell degeneration, vascular congestion/hemorrhage, and inflammatory cell infiltration. These parameters were evaluated using semi-quantitative histopathological scoring based on severity (0: none, 1: mild, 2: moderate, 3: severe). Histopathological evaluations were performed by investigators blinded to the group allocation.

### Follicle Counting

For follicle quantification, serial sections obtained from the entire right ovary were analyzed. To avoid double counting of the same follicle across adjacent serial sections, only follicles with clearly visible oocyte nuclei were included in the analysis. Follicle counts were calculated per ovary and used as independent biological replicates for statistical analysis. The numbers of primordial, primary, secondary, Graafian, atretic, and cystic follicles, as well as corpora lutea, were recorded according to previously established morphological criteria described in the literature (15).

### TUNEL assay

Apoptotic cells in oocytes and granulosa cells were detected using the MyBiosource TUNEL Assay Kit (Cat. No: MBS2557027), following the manufacturer’s instructions. For each animal, three centrally located ovarian sections were evaluated under identical microscopic conditions. TUNEL-positive cells were examined under a light microscope, and the apoptotic index was calculated as the ratio of TUNEL-positive cells to the total number of cells across all follicle types (16). TUNEL evaluations were independently performed by two investigators blinded to the experimental groups.

### Immunohistochemistry

Immunohistochemical staining was performed on central ovarian sections from each group to determine the localization of StAR, 3β-HSD, aromatase, CBS, and CTH proteins. For each animal, three centrally located ovarian sections were evaluated under identical microscopic conditions, and representative non-overlapping fields were analyzed. After deparaffinization, antigen retrieval and blocking of endogenous peroxidase activity, sections were incubated overnight at 4 °C with the following primary antibodies: StAR polyclonal antibody (1:100, Invitrogen, Cat. No: PA5-106859, RRID: AB_2854523), 3β-HSD polyclonal antibody (1:500, Invitrogen, Cat. No: PA5-27791, RRID: AB_2545267), aromatase polyclonal antibody (1:200, Invitrogen, Cat. No: PA5-86466, RRID: AB_2803244), CTH polyclonal antibody (1:100, Invitrogen, Cat. No: PA5-29725, RRID: AB_2547199), and CBS polyclonal antibody (1:200, Invitrogen, Cat. No: PA5-94923, RRID: AB_2806729). The next day, sections were treated with biotinylated secondary antibodies and streptavidin-peroxidase complex. Visualization was achieved using 3,3’-diaminobenzidine (DAB) chromogen, followed by counterstaining with hematoxylin. The intensity and distribution of staining were semi-quantitatively assessed using the H-score method calculated as: H-Score = ∑i Pi (i + 1), where Pi represents the percentage of positive cells at each intensity level and i indicates the staining intensity (1: weak, 2: moderate, 3: strong) (17, 18). Immunohistochemical evaluations were independently performed by two investigators blinded to the experimental groups.

### RNA isolation and quantitative real-time qPCR

Total RNA was isolated from ovarian tissues using the Direct-zol™ RNA MiniPrep Plus Kit (Zymo Research, R2072). RNA integrity and purity were confirmed using NanoDrop^®^ spectrophotometry (A260/280 ≈ 2.0, A260/230 > 1.8). cDNA synthesis was performed using 200 ng of total RNA, and 1 µL cDNA was used for each RT-qPCR reaction. Primer sequences used in the study are presented in **Table 1**. RT-qPCR amplification was performed with an initial denaturation step at 95 °C for 30 s, followed by 45 cycles of denaturation at 95 °C for 15 s, annealing at 52 °C for 20 s, and extension at 60 °C for 20 s. Melt-curve analysis was conducted to confirm primer specificity and exclude primer-dimer formation. Relative expression levels of target genes were calculated using the 2^–ΔΔCT^ method with β-Actin as the internal reference gene due to its stable expression across experimental groups.

**Table 1:** Primers for Cth, Cbs, CYP19A1, 3β-HSD, StAR and β-Actin used in qRT-PCR.

Primer ID	Sequence (5´-3´)
Cth_F	AAAGCACTGTTTGACCTTCG
Cth_R	GCCTCCATACACTTCATCCA
Cbs_F	CTCACATCTCCCTCCTTTCC
Cbs_R	AGGCACTCATTCCAGGAAAA
CYP19A1_F	GTGTTGAGGGTAAGTGGGAA
CYP19A1_R	GGAGCACGAACTGAGAGTAA
3β-HSD_F	TTGGTGCAGGAGAAAGAACT
3β-HSD_R	TAACATTGTCACCTTGGCCT
StAR_F	AACATGAAAGGACTGAGGCA
StAR_R	AGTGTTGCTTCCAGTTGAGA
β-Actin_F	TCTTCCAGCCTTCCTTCCTG
β-Actin_R	CACACAGAGTACTTGCGCTC

### Statistical analysis

The statistical analyses were conducted using GraphPad Prism version 10.0.2 software. The data are expressed as mean ± standard deviation. The normal distribution of the data was assessed using the Shapiro-Wilk normality test. For data conforming to normal distribution, a one-way analysis of variance (ANOVA) followed by Tukey’s multiple comparison test was performed. For data not conforming to normal distribution, the Kruskal-Wallis test followed by Dunn’s multiple comparison test was used for evaluation. p <0.05 values were considered statistically significant.

## Results

### Estrous cyclicity and serum steroid hormone levels in a DHEA-induced rat model of PCOS following NaHS administration

**Figure 2:**
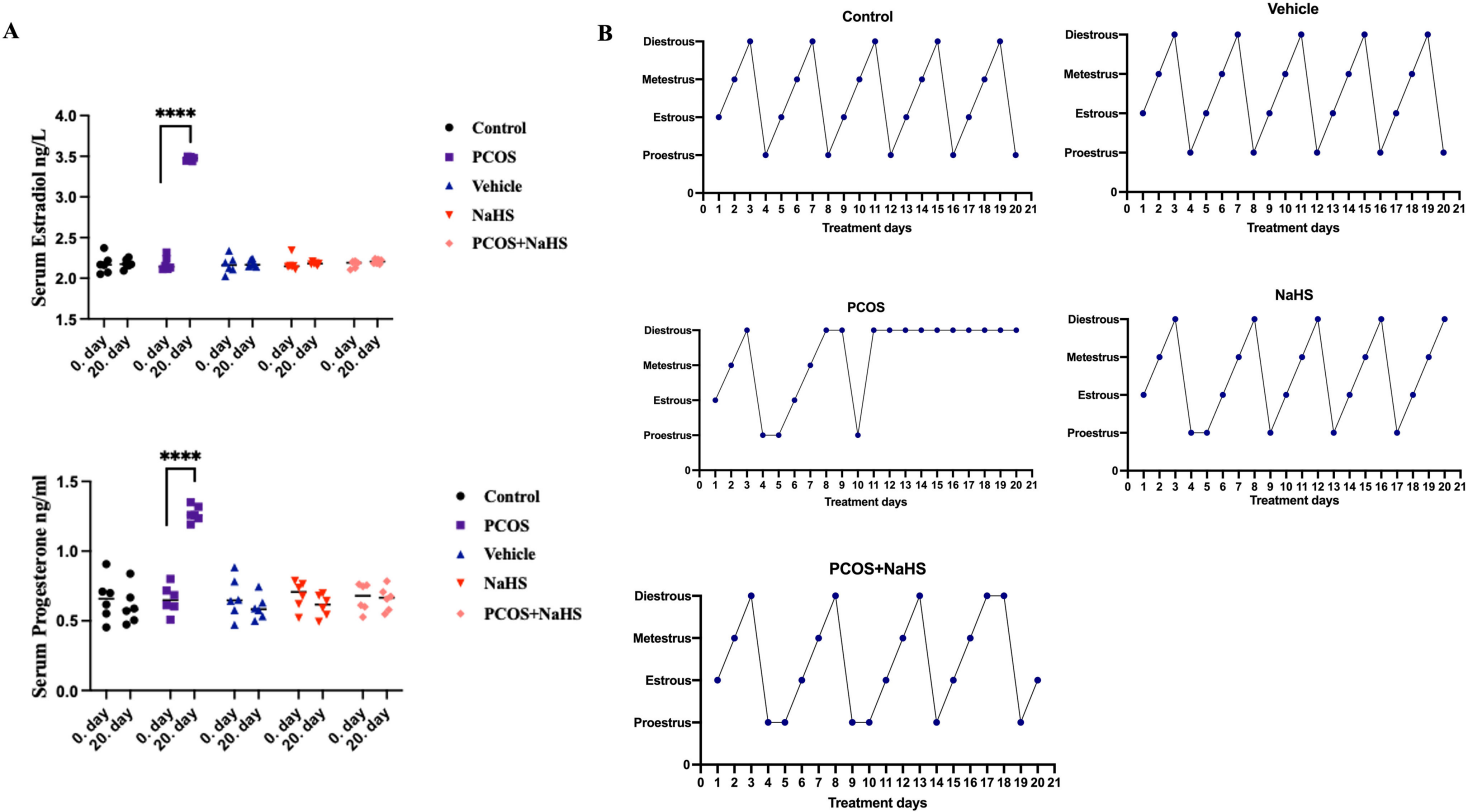
**(A)** Comparison of serum E_2_ and progesterone levels between groups before and after the experiment. ****p<0.0001. **(B)** Estrous cycle changes in representative rats from 5 groups.

Following DHEA administration, rats in the PCOS group exhibited increased serum E_2_ and progesterone levels compared with baseline values. Serum E_2_ levels increased from 2.178 ± 0.083 to 3.471 ± 0.024, while progesterone levels increased from 0.654 ± 0.102 to 1.269 ± 0.057 following DHEA administration (both p<0.0001). Rats in the control, vehicle, and NaHS groups displayed regular estrous cycles with consistent phase durations throughout the observation period. In contrast, the PCOS group showed an irregular estrous cycle pattern characterized by prolonged diestrus and the absence of transitions to proestrus, estrus, or metestrus phases. In the PCOS+NaHS group, the reappearance of all estrous cycle stages was observed, and the overall cyclic pattern approached that of the control groups (**Figure 2**).

### Follicular development and ovarian morphology in a DHEA-induced PCOS rat model following NaHS administration

Histological examination revealed normal ovarian architecture in the control and vehicle groups, characterized by follicles at various stages of development and abundant corpora lutea. In contrast, ovaries from the PCOS group exhibited multiple cystic follicles accompanied by disrupted granulosa cell layers, oocyte degeneration, stromal edema, and a reduced number of corpora lutea.

**Figure 3:**
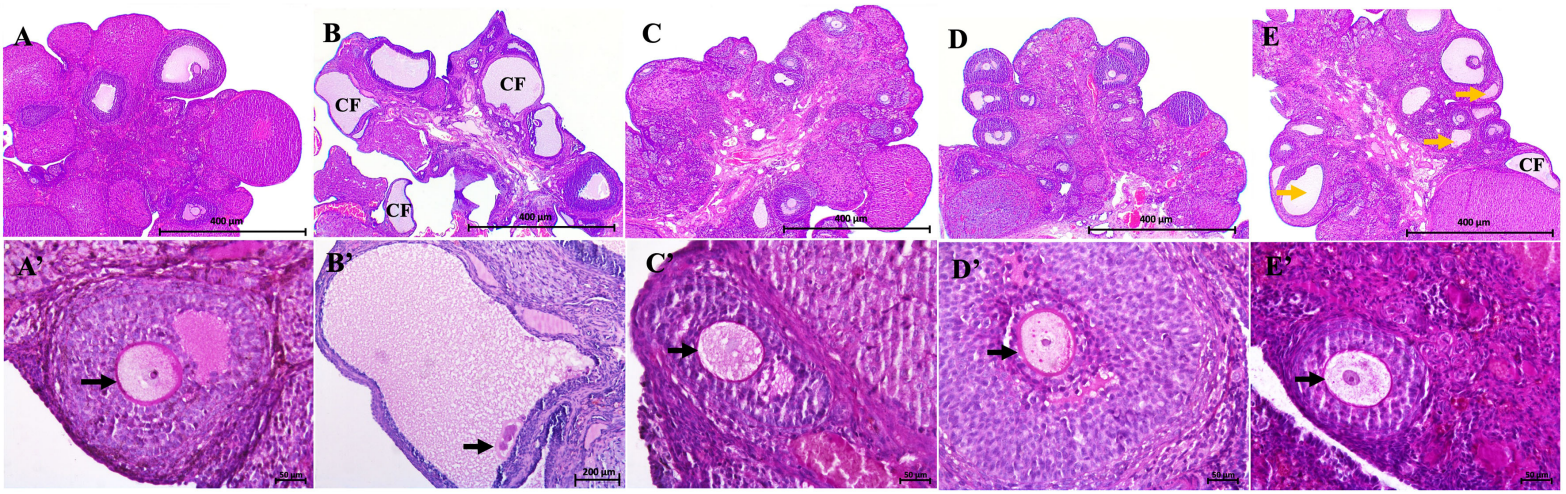
Representative ovarian sections stained with H&E **(A–E)** and PAS **(A’–E’)** across experimental groups: Control **(A, A’)**, PCOS **(B, B’)**, Vehicle **(C, C’)**, NaHS **(D, D’)**, and PCOS + NaHS **(E, E’)**. CF: cystic follicle. Yellow arrow: atretic follicle. Black arrow: zona pellucida. Scale bars: 400 μm (×4), 50 μm (×40).

In the PCOS+NaHS group, ovarian sections showed fewer cystic follicles and the presence of corpora lutea, with an overall morphological appearance comparable to that observed in the control groups. PAS staining demonstrated preservation of zona pellucida integrity in the PCOS+NaHS group, similar to that of the control ovaries (**Figure 3**).

**Figure 4:**
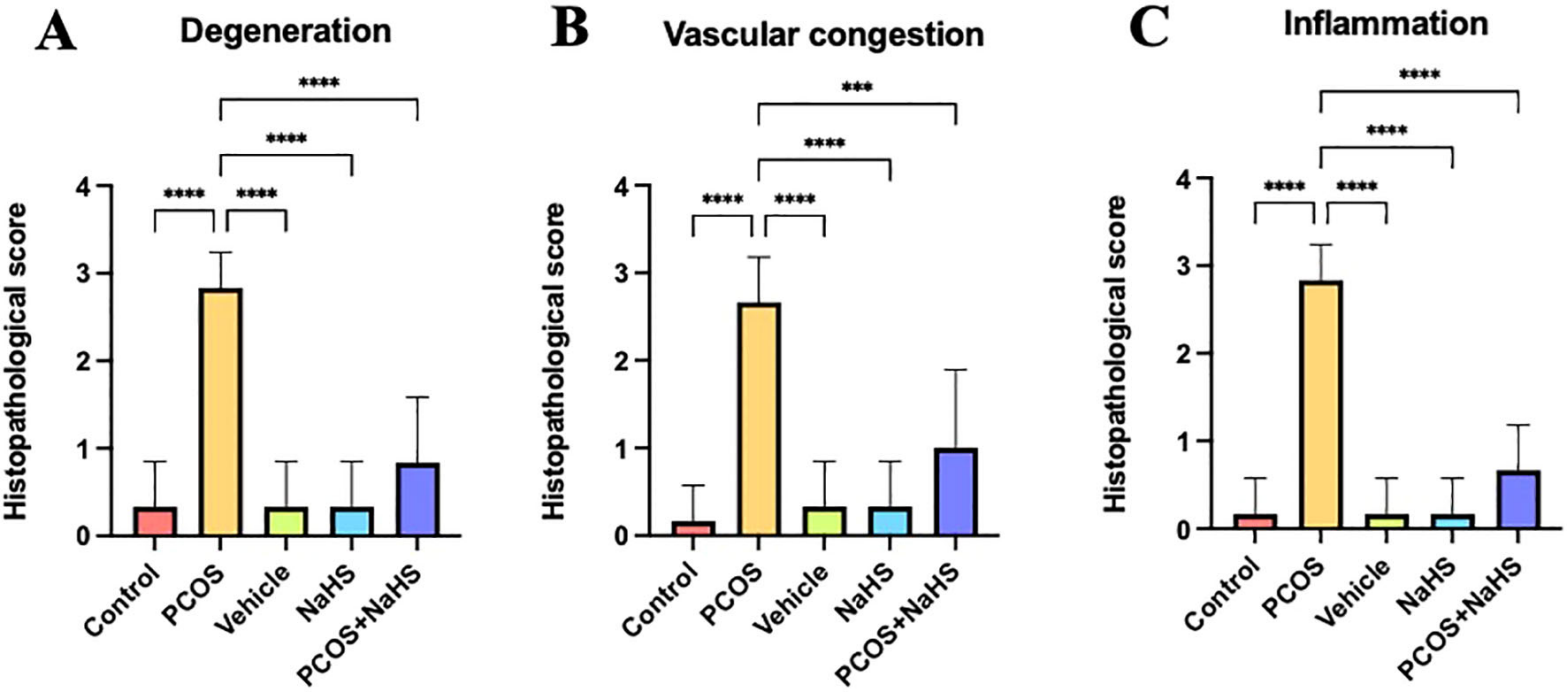
Histopathological score graph for the groups. ****p<0.0001, ***p<0.0005. **(A)** Degeneration score. **(B)** Vascular congestion score. **(C)** Inflammation score.

In addition to the descriptive histological evaluation and quantitative follicle analysis, semi-quantitative histopathological scoring was performed. Follicular degeneration, vascular congestion, and inflammatory cell infiltration scores were significantly higher in the PCOS group compared with all other groups (p < 0.0001). These scores were markedly reduced in the PCOS+NaHS group and were not statistically different from those observed in the control, vehicle, or NaHS groups (**Figure 4**).

**Figure 5:**
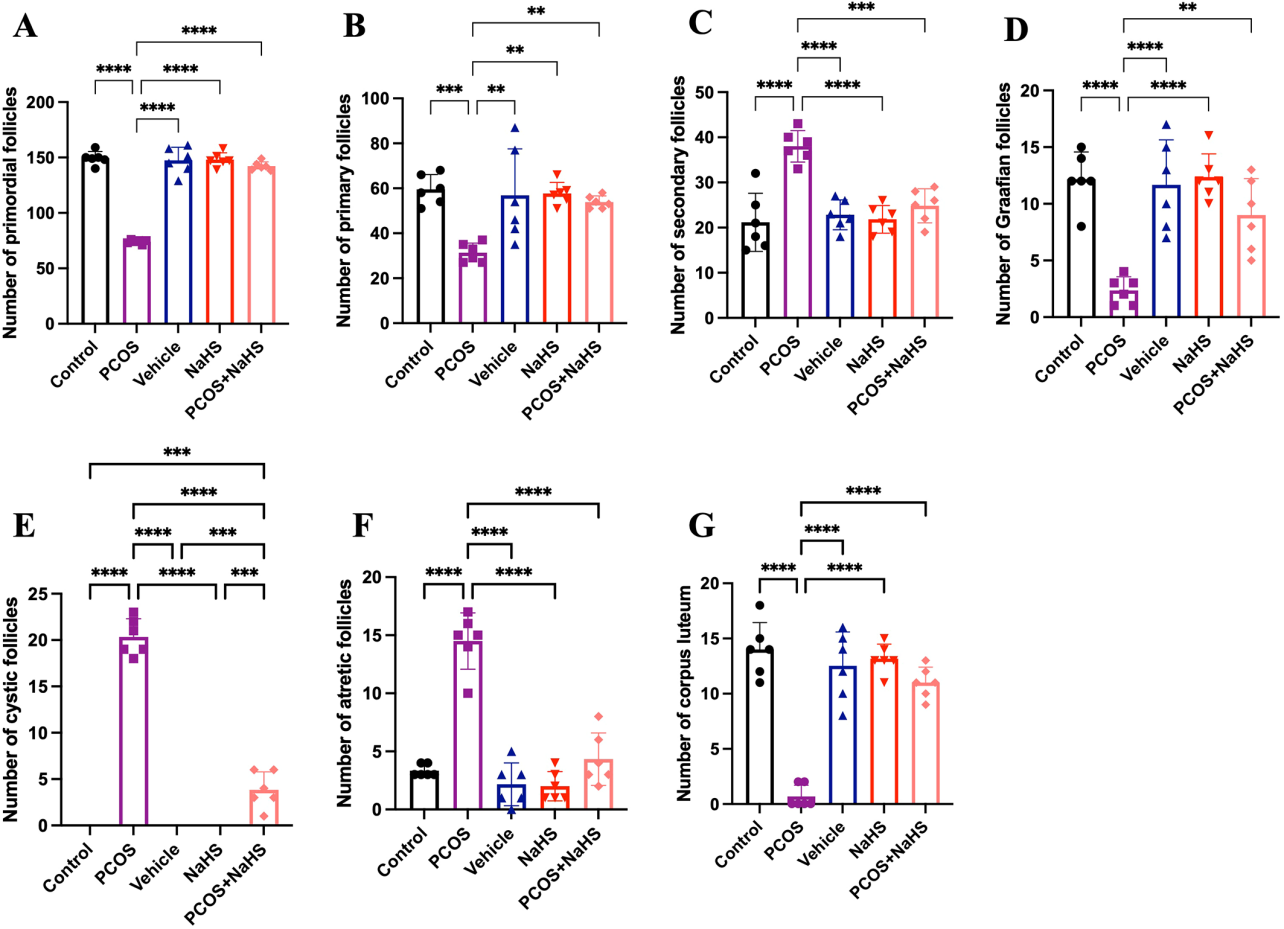
Comparison of ovarian follicular development and corpus luteum counts among experimental groups. **(A–D)** Stage-specific follicle quantification demonstrated a significant reduction in primordial, primary, and Graafian follicles, accompanied by an increase in secondary follicles in the PCOS group compared with controls (****p < 0.0001; ***p ≤ 0.006). NaHS treatment partially restored follicle numbers, particularly at the primary and Graafian stages (**p ≤ 0.0025). **(E, F)** The PCOS group exhibited a marked elevation in cystic and atretic follicles (****p < 0.0001) and a significant decrease in corpus luteum counts (****p < 0.0001). NaHS administration reduced degenerative follicle formation and restored corpus luteum numbers toward control levels (***p ≤ 0.0001; **p ≤ 0.0025). **(G)** Corpus luteum counts.

Quantitative follicle analysis revealed significant reductions in primordial, primary, and Graafian follicle counts in the PCOS group compared with the control, vehicle, and NaHS groups (primordial: 74.0 ± 1.8 vs. 149.3 ± 6.1, 147.3 ± 11.9, and 148.0 ± 6.3; primary: 31.3 ± 4.3 vs. 59.5 ± 6.6, 56.8 ± 20.7, and 57.7 ± 5.0; Graafian: 2.3 ± 1.2 vs. 12.2 ± 2.4, 11.7 ± 4.0, and 12.3 ± 2.1, respectively; p<0.0001). In contrast, secondary, cystic, and atretic follicle counts were significantly increased in the PCOS group (secondary: 38.0 ± 3.5 vs. 21.2 ± 6.4, 22.8 ± 3.3, and 21.8 ± 3.1; cystic: 20.3 ± 2.0 vs. 0, 0, and 0; atretic: 14.5 ± 2.4 vs. 3.3 ± 0.5, 2.2 ± 1.8, and 2.0 ± 1.3, respectively; p<0.0001). Corpus luteum counts were markedly decreased in the PCOS group (0.7 ± 1.0 vs. 14.0 ± 2.4, 12.5 ± 3.1, and 13.2 ± 1.3, respectively; p<0.0001). In the PCOS+NaHS group, follicle distribution and corpus luteum counts approached the values observed in the control, vehicle, and NaHS groups (**Figure 5**).

### Apoptotic activity in ovarian tissue in a DHEA-induced PCOS rat model following NaHS administration

**Figure 6:**
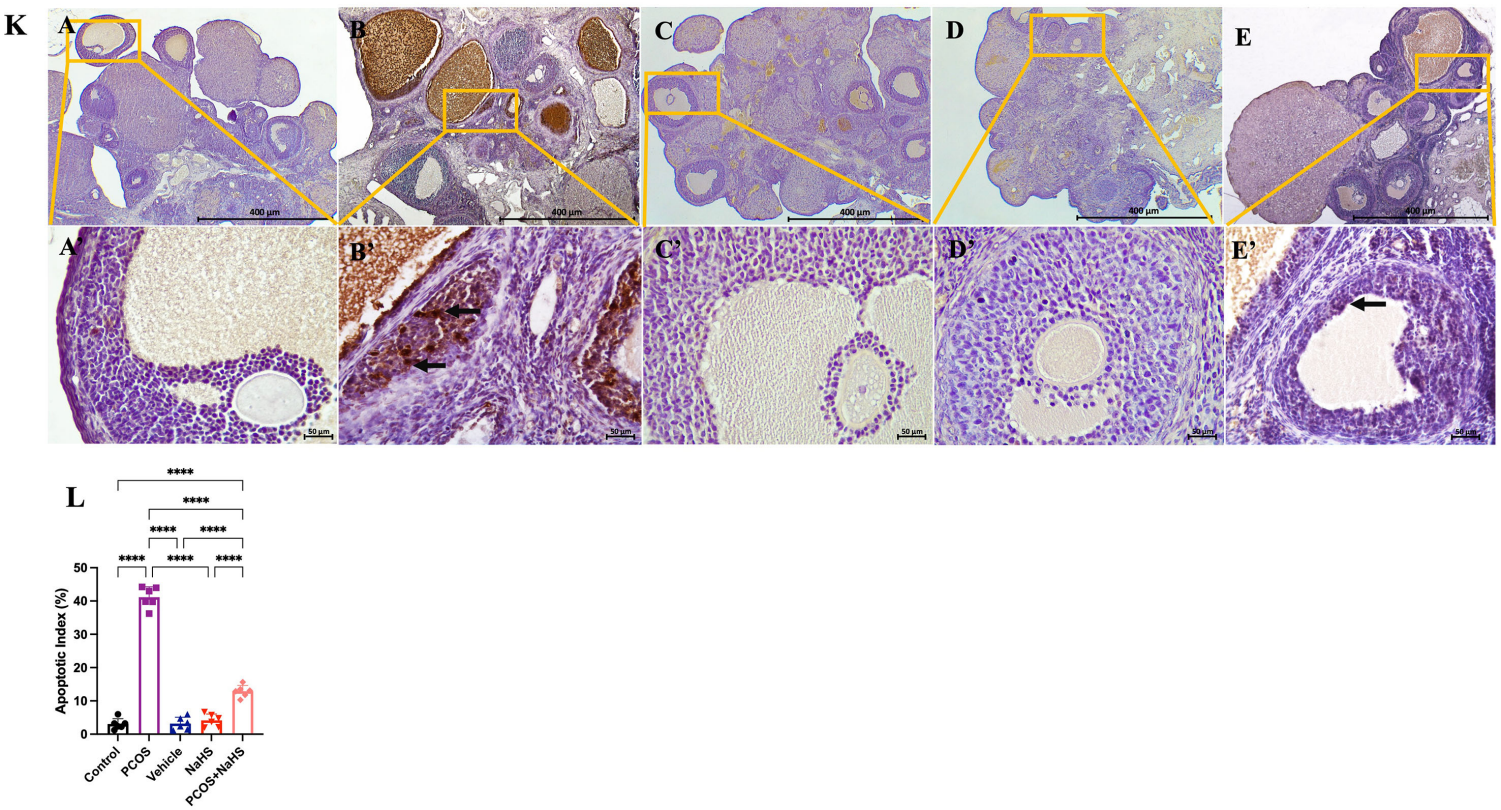
**(K) **TUNEL assay-based detection of apoptotic follicles in ovarian tissues across experimental groups. Control **(A, A’)**, PCOS **(B, B’)**, Vehicle **(C, C’)**, NaHS **(D, D’)**, PCOS+NaHS **(E, E’)**. TUNEL-positive cells are indicated by black arrows. Scale bars: 400 μm (×4) and 50 μm (×40). **(L)** Graphical representation of apoptotic index across experimental groups. ****p<0.0001.

The PCOS group exhibited a significantly higher apoptotic index compared with the control, vehicle, NaHS, and PCOS+NaHS groups (p<0.0001). In the PCOS+NaHS group, the apoptotic index was partially reduced compared with the untreated PCOS group (p<0.0001); however, it remained significantly higher than the values observed in the control, vehicle, and NaHS groups (p<0.0001) (**Figure 6**).

### Immunohistochemical expression of StAR, 3β-HSD, and CYP19A1 proteins in the ovaries of DHEA-induced PCOS rats following NaHS administration

Immunohistochemical analysis revealed cytoplasmic StAR immunoreactivity in the theca interna, interstitial cells, and granulosa lutein cells of the control, vehicle, NaHS, and PCOS+NaHS groups. In contrast, ovaries from the PCOS group exhibited significantly higher StAR staining intensity in granulosa, theca, and stromal cells (p<0.0001), whereas cystic follicles showed relatively weaker thecal staining.

Similarly, 3β-HSD immunoreactivity was localized predominantly in the theca and interstitial cells across all groups. Quantitative analysis demonstrated a significantly increased staining intensity in the PCOS group compared with the other groups (p<0.0001). In addition, granulosa cells of cystic follicles exhibited increased 3β-HSD immunoreactivity in the PCOS group, while minimal staining was observed in the control, vehicle, NaHS, and PCOS+NaHS groups.

**Figure 7:**
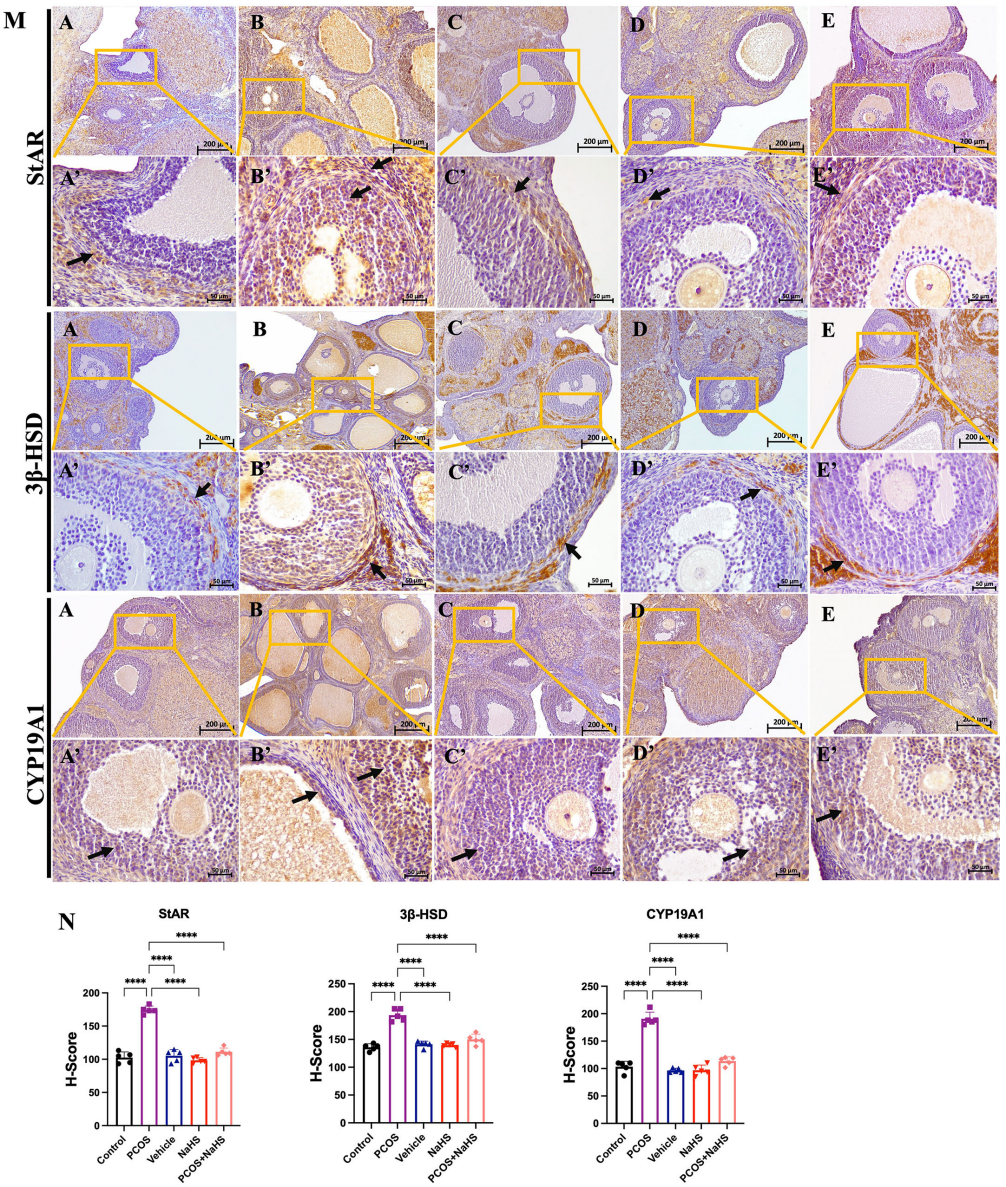
**(M)** Immunohistochemical localization of StAR, 3β-HSD and CYP19A1 in ovarian tissues across experimental groups. Control **(A, A’)**, PCOS **(B, B’)**, Vehicle **(C, C’)**, NaHS **(D, D’)**, PCOS+NaHS **(E, E’)**. Black arrows indicate StAR-, 3β-HSD-, CYP19A1-positive immunoreactivity. Scale bars: 200 μm (×10) and 50 μm (×40). **(N)** H-score analysis of StAR, 3β-HSD and CYP19A1 expression in ovarian tissues across experimental groups ****p < 0.0001.

CYP19A1 expression was detected in the granulosa cells of developing and Graafian follicles, as well as in corpus luteum cells, but was absent in primordial follicles. CYP19A1 immunoreactivity was significantly higher in the granulosa cells of the PCOS group (p<0.0001), whereas no significant differences were observed among the control, vehicle, NaHS, and PCOS+NaHS groups (**Figure 7**).

### CBS and CTH protein expression in the ovaries of DHEA-induced pcos rats following NaHS administration

**Figure 8:**
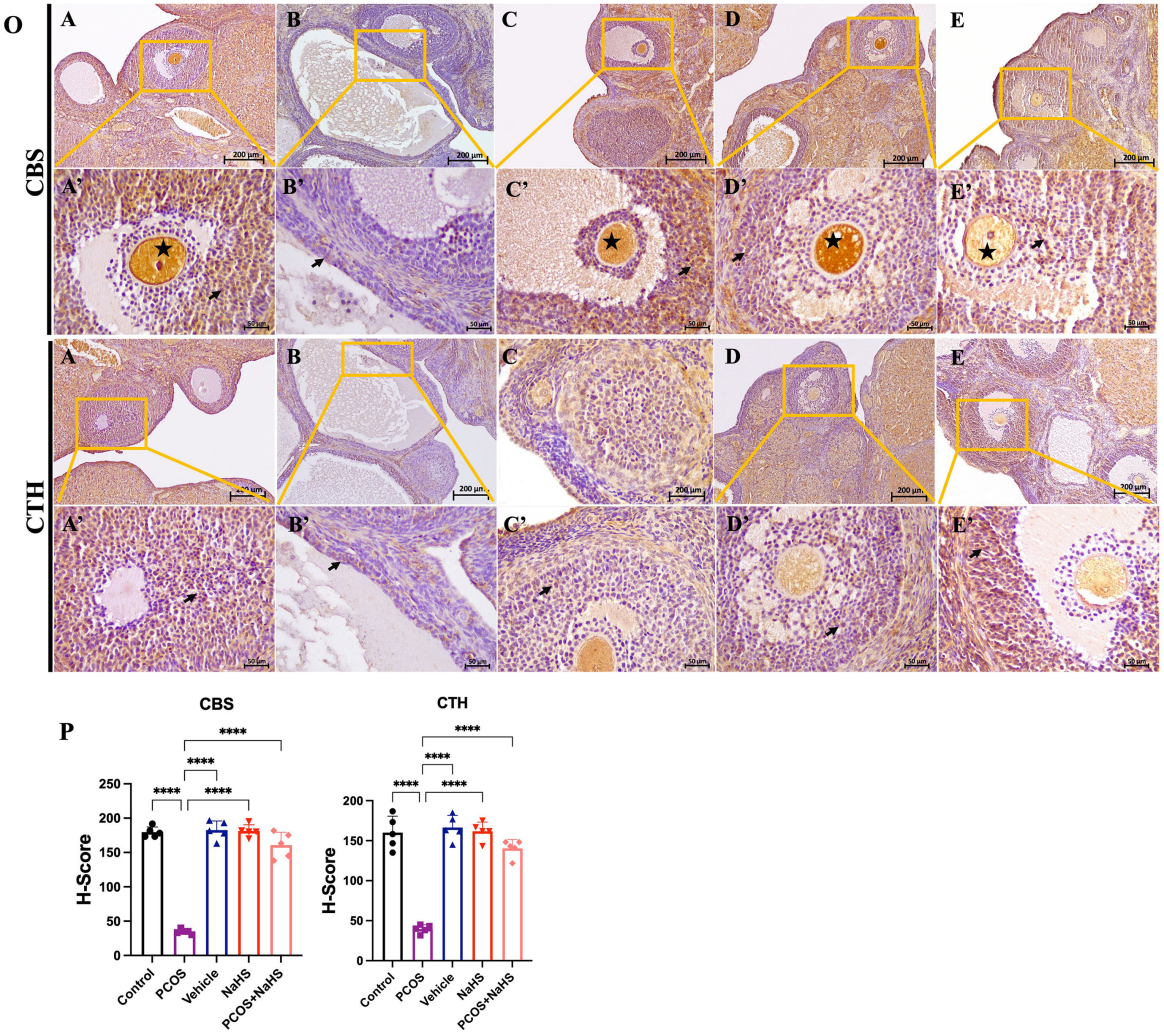
**(O)** Immunohistochemical localization of CBS and CTH in ovarian tissues across experimental groups. Control **(A, A’)**, PCOS **(B, B’)**, Vehicle **(C, C’)**, NaHS **(D, D’)**, PCOS+NaHS **(E, E’)**. Star: Oocyte. Black arrows indicate CBS- or CTH-positive immunoreactivity. Scale bars: 200 μm (×10) and 50 μm (×40). **(P)** H-score analysis of CBS and CTH expression in ovarian tissues across experimental groups ****p < 0.0001.

CBS immunoreactivity was detected in the cytoplasm of granulosa cells of developing follicles and oocytes, as well as in theca, interstitial, smooth muscle, and luteal cells across all groups. In the PCOS group, CBS expression was markedly reduced in granulosa, theca, and interstitial cells, and no detectable staining was observed in oocytes. Quantitative H-score analysis confirmed a significant decrease in CBS expression in the PCOS group compared with all other groups (p<0.0001) (**Figure 8**).

Similarly, CTH immunoreactivity was observed in granulosa, theca, interstitial, and luteal cells in all groups. In the PCOS group, CTH staining intensity was lower in oocytes and comparatively weaker than that observed in control groups (p<0.0001). In the PCOS+NaHS group, CBS and CTH immunoreactivity levels were comparable to those observed in the control groups (**Figure 8**).

### mRNA expression levels of steroidogenic enzymes and H_2_S-producing enzymes in DHEA-induced PCOS rats

StAR mRNA expression was significantly increased in the PCOS group compared with the control, vehicle, and NaHS groups (1.592 ± 0.090 vs. 0.851 ± 0.166, 0.437 ± 0.030, and 0.655 ± 0.310, respectively; p<0.0006). Similarly, 3β-HSD mRNA expression was significantly elevated in the PCOS group (2.873 ± 0.644 vs. 0.931 ± 0.161, 0.382 ± 0.176, and 0.402 ± 0.292, respectively; p<0.0005). CYP19A1 mRNA expression was also markedly increased in the PCOS group (14.73 ± 4.94 vs. 1.001 ± 0.040, 0.883 ± 0.806, and 1.203 ± 0.647, respectively; p<0.0001). In contrast, the expression levels of these steroidogenic enzymes in the PCOS+NaHS group were markedly reduced compared with the PCOS group and approached the levels observed in the control, vehicle, and NaHS groups.

**Figure 9:**
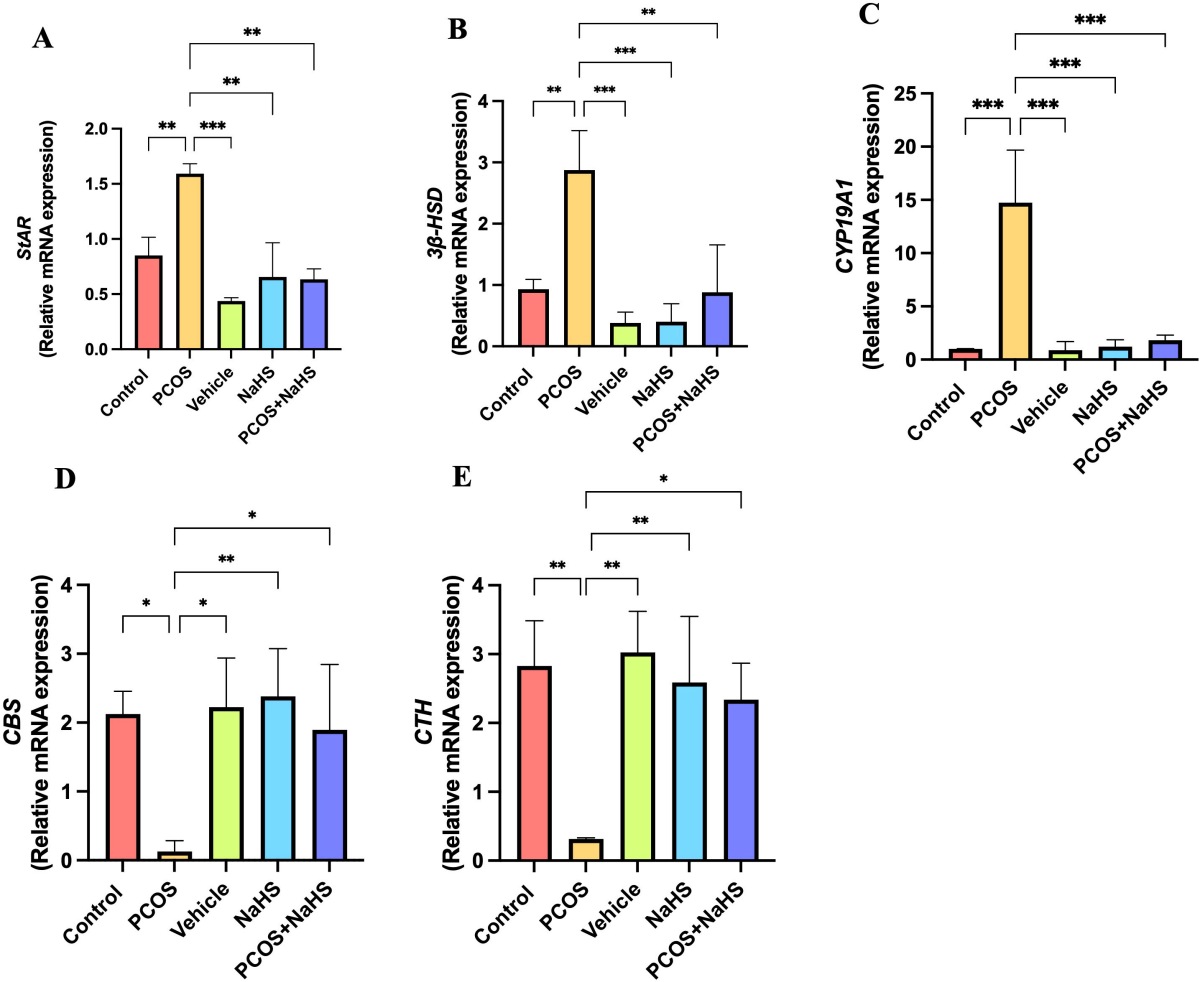
Relative mRNA expression levels of steroidogenic genes (StAR, 3β-HSD, CYP19A1) and H_2_S-producing enzymes (CBS, CTH) in experimental groups. **(A)** StAR: Control vs PCOS (p=0.0091); PCOS vs Vehicle (p=0.0004); PCOS vs NaHS (p=0.0024); PCOS vs PCOS+NaHS (p=0.0012). **(B)** 3β-HSD: Control vs PCOS (p=0.0041); PCOS vs Vehicle (p=0.0006); PCOS vs NaHS (p=0.0007); PCOS vs PCOS+NaHS (p=0.0034). **(C)** CYP19A1: The PCOS group showed significantly higher expression compared with the Control, Vehicle, and NaHS groups (p < 0.002), as well as the PCOS+NaHS group (p = 0.0003). **(D)** CBS: Control vs PCOS (p=0.0212); PCOS vs Vehicle (p=0.0158); PCOS vs NaHS (p=0.0100); PCOS vs PCOS+NaHS (p=0.0420). **(E)** CTH: Control vs PCOS (p=0.0046); PCOS vs Vehicle (p=0.0026); PCOS vs NaHS (p=0.0090); PCOS vs PCOS+NaHS (p=0.0188). Statistical significance was indicated as follows: *p < 0.05, **p < 0.01, *p < 0.001.

In contrast, the mRNA expression levels of CBS and CTH were significantly decreased in the PCOS group compared with the control, vehicle, and NaHS groups. CBS expression was reduced to 0.129 ± 0.155 compared with 2.124 ± 0.330, 2.225 ± 0.715, and 2.382 ± 0.692 in the control, vehicle, and NaHS groups, respectively (p<0.0083). Similarly, CTH expression decreased to 0.315 ± 0.018 in the PCOS group compared with 2.828 ± 0.657, 3.025 ± 0.597, and 2.587 ± 0.962 in the control, vehicle, and NaHS groups, respectively (p<0.0024). In the PCOS+NaHS group, CBS and CTH expression levels increased to 1.896 ± 0.948 and 2.338 ± 0.529, respectively, approaching the values observed in the control, vehicle, and NaHS groups (**Figure 9**).

## Discussion

The present study demonstrates that pharmacological supplementation with the hydrogen sulfide donor, NaHS was associated with modulation of steroidogenic enzyme expression, apoptotic activity, and follicular architecture in a DHEA-induced rat model of PCOS. Specifically, NaHS administration attenuated DHEA-induced upregulation of StAR, 3β-HSD, and CYP19A1, reduced apoptotic indices, and partially restored the expression of CBS and CTH within ovarian tissue. These findings support a potential interaction between H_2_S-related pathways and ovarian dysfunction in experimental PCOS. Among androgen-induced rodent models, DHEA administration is widely used due to its ability to reproduce key reproductive features of PCOS, including anovulation, cystic follicle formation, and steroidogenic dysregulation. Although no animal model fully recapitulates the heterogeneous clinical spectrum of human PCOS, the DHEA model provides a controlled experimental framework to investigate ovarian molecular alterations (19).

Hormonal imbalances arising from dysfunction of the hypothalamic–pituitary–ovarian (HPO) axis constitute the basis of impaired folliculogenesis in PCOS. Enhanced secretion of gonadotrophin releasing hormone (GnRH) stimulates increased LH release from the adenohypophysis, thereby promoting increased androgen production by theca cells (20). This process leads to the overexpression of key steroidogenic enzymes, such as CYP17A1 and StAR (21, 22), ultimately aggravating hyperandrogenism. In addition, insulin resistance further exacerbates hyperandrogenism by premature luteinization of granulosa cells and enhanced steroid production (23). Notably, the increased progesterone synthesis in small follicles and the upregulation of LH receptor expression during the early stages of folliculogenesis contribute to premature luteinization (24). Collectively, these mechanisms lead to follicular arrest, anovulation and disrupted ovarian function. These endocrine alterations are expected to manifest as functional disturbances in estrous cyclicity and ovulatory capacity.

Although serum progesterone levels were elevated following chronic DHEA administration, this finding should be interpreted within the context of DHEA-induced endocrine dysregulation rather than coordinated luteal function. In the present study, hormone measurements reflected chronic androgen exposure and ovarian steroidogenic imbalance rather than cycle phase-dependent physiological secretion. Premature luteinization of granulosa cells, characterized by early LH receptor expression and increased progesterone synthesis in small follicles, has been described in both clinical and experimental PCOS models (25, 26). Therefore, the elevated progesterone levels observed in the PCOS group are more likely to reflect dysregulated granulosa cell steroidogenesis than functional ovulation. Nevertheless, a potential contribution of adrenal-derived steroids secondary to DHEA administration can not be completely excluded.

Irregular estrous cycles and anovulation are frequently observed in DHEA-induced PCOS models (26–28). In the present study, only the PCOS group exhibited a prolonged diestrus phase, confirming disruption of the normal cyclic pattern. Moreover, the absence or presence of only a few corpus luteum structures in this group supports the occurrence of anovulation, consistent with the vaginal smear findings and validating the successful establishment of the PCOS model. In addition, the DHEA-induced PCOS phenotype was further supported by histopathological findings, follicular alterations, and serum estradiol/progesterone measurements. However, additional endocrine markers including testosterone, LH, and FSH were not evaluated. The inclusion of these endocrine parameters would have provided a more comprehensive characterization of the hormonal profile of the experimental model and strengthened the overall endocrine validation of the PCOS phenotype. In contrast, preservation of regular estrous cyclicity in the NaHS-treated group suggests a modulatory influence of hydrogen sulfide on ovarian physiology. H_2_S has recently emerged as a novel regulator of ovarian function, exerting significant modulatory effects on folliculogenesis and ovulatory mechanisms. H_2_S has been shown to upregulate ovulatory genes such as AREG, EREG, and PLAT and to facilitate follicular wall rupture via activation of matrix-degrading enzymes including MMP2 and MMP9 (10). In line with these findings, normalization of estrous cycles and the distinct presence of corpus luteum structures in the PCOS+NaHS group in this study support a potential ovulation-modulating role of H_2_S.

In DHEA-induced PCOS rats, ovarian histopathology typically reveals an increased stromal area, numerous cystic follicles, evident degeneration of the zona pellucida and oocyte levels and inflammatory cell infiltration, findings that closely resemble the clinical phenotype of PCOS in humans (14, 29, 30). In the present study, NaHS treatment was associated with reduced cystic morphology and improved follicular integrity compared to untreated PCOS animals. These findings indicate a partial structural improvement following H_2_S donor administration.

Increased apoptosis in ovarian granulosa cells represents a key component of PCOS pathophysiology. In DHEA-induced experimental models, elevated apoptotic index and enhanced follicular atresia have been linked to follicular developmental arrest and progressive degeneration (31, 32). Consistent with these reports, our TUNEL analysis demonstrated a significantly increased apoptotic index in the PCOS group, paralleling the higher number of atretic follicles. Notably, in the PCOS+NaHS group, the number of atretic follicles was comparable to the other groups, whereas the apoptotic index was markedly reduced. This dissociation suggests that NaHS may suppress early molecular apoptotic signaling before overt structural degeneration becomes evident. Indeed, preantral and early antral follicles in PCOS often appear histologically preserved, with degenerative changes emerging predominantly during later antral transition, which may partly explain this discrepancy. However, apoptosis was not fully normalized following NaHS treatment, indicating that H_2_S supplementation may attenuate but not completely modulate all apoptosis-related pathways involved in PCOS pathogenesis.

The reduction in apoptotic activity following NaHS administration observed in this study aligns with the well-documented anti-apoptotic effects of H_2_S in various tissues and cell models. Deficiency of H_2_S-producing enzymes has been linked to increased cellular senescence and apoptosis, both of which have been shown to be alleviated by H_2_S supplementation (33, 34). Additionally, H_2_S has been reported to modulate key apoptotic regulators such as caspases, Bax, Bcl-2, and Nrf2, thereby maintaining cellular homeostasis (35). Taken together, these findings suggest that the reduction in apoptosis following NaHS treatment may reflect a potential modulatory influence of H_2_S on ovarian tissue. However, these molecular pathways were not directly evaluated in the present study and should therefore be interpreted as potential mechanisms underlying the observed effects of NaHS treatment. Further studies investigating apoptosis-related, oxidative stress-related, and downstream molecular signaling pathways will be necessary to clarify the precise molecular actions of H_2_S in PCOS.

Based on the pattern of apoptotic reduction observed in our study, the persistence of a higher apoptotic index compared to controls reflects an incomplete rescue rather than a complete restoration of follicular integrity. Nevertheless, the involvement of H_2_S-independent apoptotic pathways cannot be excluded and may contribute to the residual apoptotic activity observed in the PCOS+NaHS group. Accordingly, future studies incorporating additional apoptotic markers, particularly caspase activation and downstream signaling pathways, will be essential to further clarify H_2_S-dependent and -independent mechanisms involved in ovarian apoptosis.

Alterations in the expression of steroidogenic enzymes are central to the endocrine dysfunction observed in PCOS. Previous studies have demonstrated that DHEA administration increases the gene and protein expression levels of key enzymes such as StAR, 3β-HSD, CYP11A1, and CYP19A1 in granulosa cells, leading to elevated levels of estradiol, progesterone, and androgens. These hormonal changes contribute to hyperandrogenism and impaired follicular development (25, 27, 36–38). PCOS-associated alterations in aromatase activity have been reported inconsistently across experimental studies. Several DHEA-induced PCOS models have demonstrated increased CYP19A1 expression following chronic androgen exposure (12, 25, 38, 39). Excessive androgen stimulation in granulosa cells may induce compensatory upregulation of aromatase activity in an attempt to enhance androgen-to-estrogen conversion. However, despite this increase, persistent steroidogenic dysregulation may still impair normal follicular maturation and contribute to cystic follicle formation. Consistent with these reports, our study revealed a significant increase in both immunoreactivity and mRNA expression levels of these enzymes in the DHEA-induced PCOS group. Interestingly, NaHS treatment markedly suppressed this steroidogenic overactivation. In the PCOS+NaHS group, both gene and protein expression levels of StAR, 3β-HSD, and CYP19A1 were significantly reduced compared to PCOS group and were comparable to those observed in the control groups. These findings suggest that NaHS treatment was associated with reduced expression of selected steroidogenic markers, suggesting partial attenuation of DHEA-induced steroidogenic dysregulation. To the best of our knowledge, our findings provide initial experimental evidence suggesting that H_2_S may contribute to the regulation of steroidogenic homeostasis in a DHEA-induced PCOS model. Previous studies investigating H_2_S donor administration in experimental PCOS models have primarily focused on oxidative stress status and alterations in ovarian H_2_S-producing enzymes (11). In contrast, the present study additionally evaluated the expression of key steroidogenic enzymes, including StAR, 3β-HSD, and CYP19A1, at both immunohistochemical and mRNA expression levels. Furthermore, ovarian histopathology, estrous cyclicity, and apoptotic activity were assessed simultaneously within the same experimental model. Collectively, these findings provide a more integrated evaluation of the potential association between H_2_S-related pathways and steroidogenic dysregulation in experimental PCOS.

In addition, expression of CBS and CTH—the key enzymes involved in endogenous H_2_S biosynthesis— has been previously reported in the uterus, placenta, fallopian tubes, and oocytes (6, 7, 9, 40, 41). In our study, both CBS and CTH were localized in all follicular stages of the rat ovary, specifically within granulosa and theca cells, interstitial areas, and corpus luteum. Notably, for the first time, positive immunoreactivity for these enzymes was also detected in the oocyte cytoplasm, with CBS exhibiting a stronger signal than CTH at the oocyte level. Consistent with previous findings, DHEA administration resulted in a reduction of CBS and CTH expression, which may suppress endogenous H_2_S production. Conversely, in the PCOS+NaHS group, both enzymes showed a significant increase in expression levels. The restoration of CBS and CTH expression suggests that NaHS treatment not only replenishes H_2_S bioavailability but also re-establishes the balance of H_2_S-generating system within ovarian tissue, which may contribute to ovarian protection and improved follicular integrity. These findings suggest that NaHS treatment may contribute not only to increased H_2_S bioavailability but also to the re-establishment of the endogenous H_2_S-generating system within ovarian tissue, potentially supporting ovarian protection and improved follicular integrity.

Despite the strengths of the present study, several limitations should be acknowledged. First, circulating androgen and gonadotropin levels, including testosterone, LH, and FSH, were not directly measured, which limits comprehensive endocrine characterization of the PCOS model. Second, ovarian hydrogen sulfide concentrations were not directly quantified; instead, CBS and CTH expression was used as an indirect indicator of endogenous H_2_S metabolism. As enzyme expression does not necessarily reflect functional H_2_S bioavailability, definitive mechanistic conclusions cannot be drawn regarding active H_2_S signaling. Furthermore, downstream molecular signaling pathways through which H_2_S may exert its ovarian effects were not investigated in detail. Finally, although estrous cycle monitoring and standardized tissue collection were implemented, the residual cycle-stage variability can not be completely excluded.

In conclusion, NaHS supplementation in a DHEA-induced PCOS rat model was associated with improvements in ovarian morphology, a reduction in apoptotic activity, and partial normalization of steroidogenic and H_2_S-producing enzyme expression. These findings suggest that hydrogen sulfide may play a modulatory role in the maintenance of ovarian homeostasis under PCOS-like conditions. The restoration of estrous cyclicity, increased corpus luteum formation, and improvement in follicular integrity observed following NaHS administration may also suggest a potential beneficial effect on ovulatory function and reproductive capacity in experimental PCOS. Although direct fertility outcomes were not evaluated in the present study, these findings indicate that modulation of H_2_S-related pathways may have translational relevance for ovarian dysfunction associated with PCOS. However, given the absence of direct H_2_S quantification and detailed mechanistic analyses, the translational implications of these results should be interpreted with caution. Future studies incorporating functional H_2_S measurements, detailed signaling pathway analyses and long-term fertility outcomes will be essential to clarify the therapeutic potential of targeting H_2_S-related pathways in PCOS.

## Data Availability

The original contributions presented in the study are included in the article/supplementary material. Further inquiries can be directed to the corresponding author/s.
